# Chronic Venous Insufficiency and Lymphedema With Papillomatosis Cutis Lymphostatica, Hyperkeratosis, and Skin Ulcers: A Case Report

**DOI:** 10.7759/cureus.35326

**Published:** 2023-02-22

**Authors:** Jayani Senanayake, Sanket Chaudhari, Rangin Haji Rahman, Sally Madanat, Frederick Tiesenga

**Affiliations:** 1 Internal Medicine, Saint James School of Medicine, Chicago, USA; 2 Surgery, Saint James School of Medicine, Chicago, USA; 3 Pediatrics, Saint James School of Medicine, Chicago, USA; 4 Medicine, Washington University of Health and Science, San Pedro, BLZ; 5 General Surgery, West Suburban Medical Center, Oak Park, USA

**Keywords:** lymphedema, papillomatosis cutis lymphostatica, proteus mirabilis, chronic venous insufficiency, hyperkeratosis

## Abstract

Chronic venous insufficiency (CVI) is a common condition characterized by lower extremity edema, discomfort, and skin changes due to venous hypertension caused by incompetent or obstructed venous valves. We report a case of chronic venous insufficiency and lymphedema with papillomatosis cutis lymphostatica, hyperkeratosis, and skin ulcers with proteus superinfection. A 67-year-old male presented to the emergency department (ED) for wound evaluation and was found to have severe hyperkeratosis, multiple ulcers with purulent discharge, and “tree bark” skin changes. Prophylactic treatment for deep vein thrombosis (DVT) was initiated, followed by successful surgical debridement. A subsequent diagnosis of *Proteus mirabilis* superinfection was treated accordingly. This report highlights the importance of adequate long-term management of chronic venous insufficiency as it may lead to serious complications.

## Introduction

Chronic venous insufficiency (CVI) is a common condition in the adult population, with a prevalence of 25%-40% and 10%-20% in females and males, respectively [1]. It is characterized by lower extremity edema, lower extremity discomfort, and accompanying skin trophic changes [2]. The risk factors for chronic venous insufficiency include advanced age, history of deep vein thrombosis (DVT), sedentary lifestyle, use of oral contraceptives, leg injury, smoking, and hypertension [2]. Physiologically, it is due to venous hypertension as a result of incompetent venous valves, valve destruction, or obstruction of the venous system [3]. Venous valves are one-way valves, possessing two cusps with edges that meet one another, promoting the antegrade flow of blood toward the heart. As such, they are crucial for preventing the backflow of blood. The valves are pushed open with the help of the skeletal muscles surrounding the veins, as blood circulates back to the heart. When skeletal muscle relaxation occurs, the valvular cusps are pushed closed. When this mechanism is impaired, retrograde blood flow ensues, resulting in blood pooling in the lower extremities. From a clinical standpoint, patients commonly present with a combination of lower extremity edema, leg discomfort, a feeling of heaviness and/or tingling in the leg, fatigue, and itching, and symptoms may improve with rest and elevation [2].

Complications are not uncommon in long-standing venous insufficiency (VI). These include venous leg ulcers, the most frequent sequela, as well as dermatitis, atrophie blanche, lipodermatosclerosis, and malignancy [4]. A common non-dermatologic complication of chronic venous insufficiency is secondary lymphedema, which is sometimes referred to as “phlebolymphedema” [5]. Damage to the lymphatic circulation is thought to occur as a result of lymphatic overload or recurrent cellulitis [5]. Papillomatosis cutis lymphostatica is a severe complication of chronic lymphedema and venous insufficiency characterized by hyperkeratotic, papillomatous, and verrucous skin lesions [6,7]. This article presents a case of a 67-year-old male with chronic venous insufficiency and secondary lymphedema with papillomatosis cutis lymphostatica, hyperkeratosis, and skin ulcers with proteus superinfection.

## Case presentation

**Figure 1:**
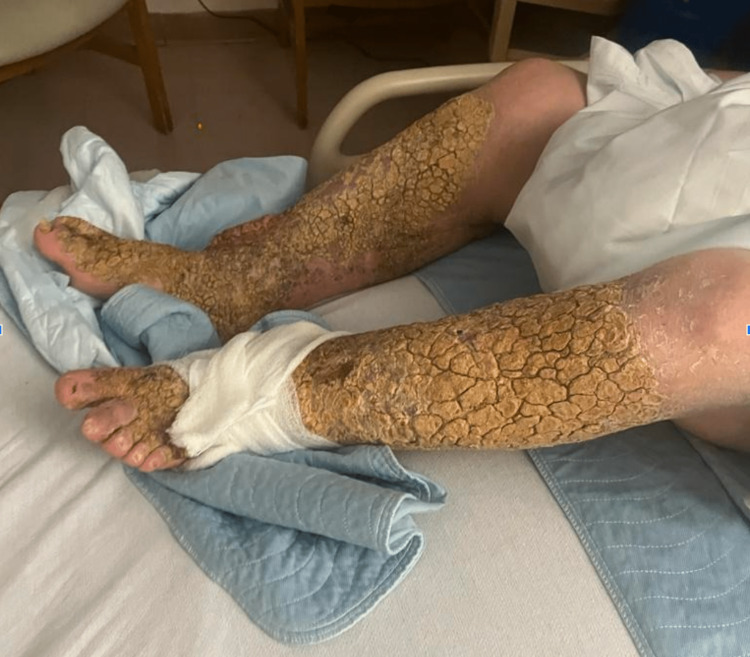
Hyperkeratosis and nodular skin growths resembling “tree bark” skin changes

**Figure 2:**
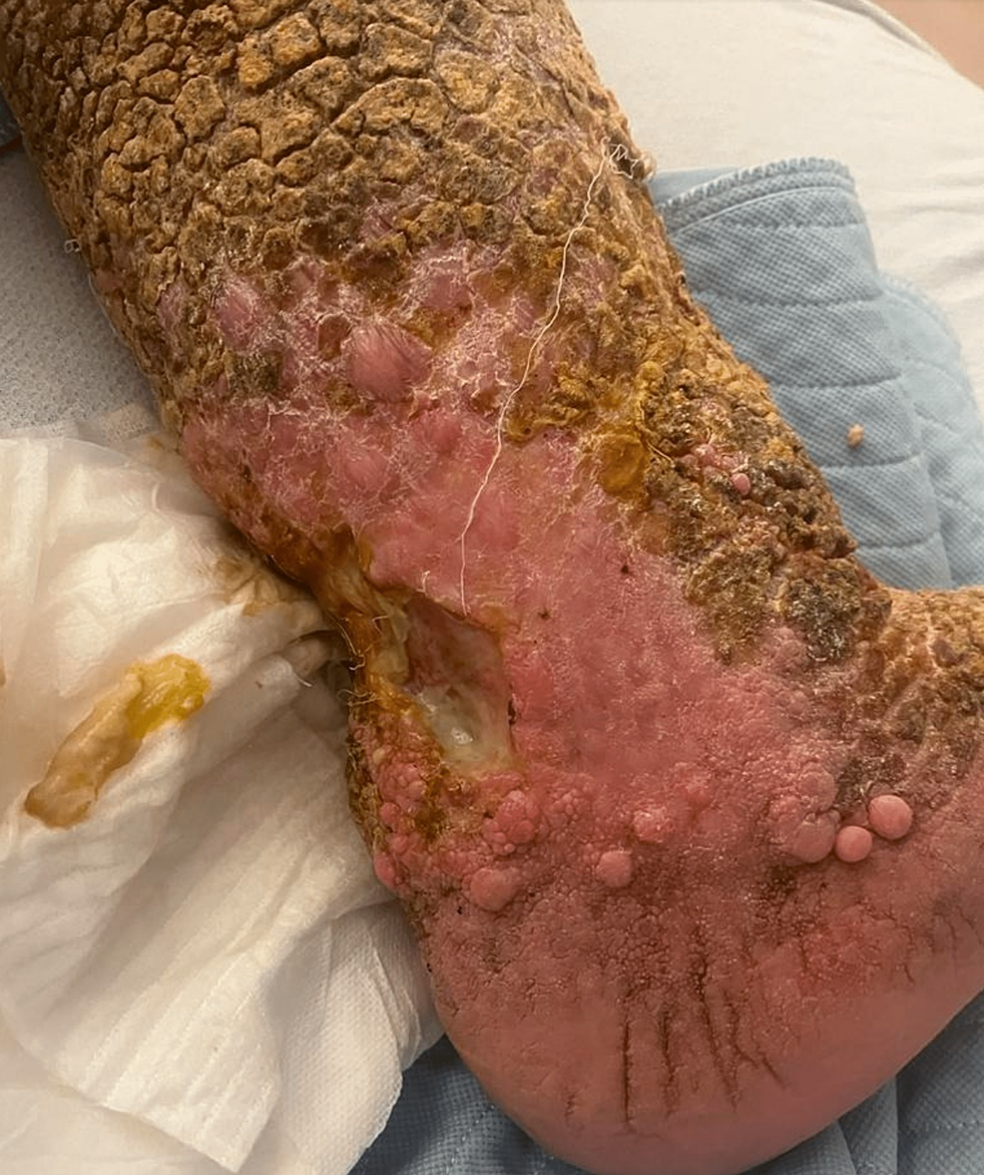
Necrotic ulcer on the left posterior ankle with purulent discharge

A 67-year-old male was brought to the emergency department (ED) from a nursing home (NH) for a wound check evaluation. According to the NH staff, the patient was uncooperative and refused wound care treatment. His past medical history is significant for type two diabetes mellitus (T2DM), chronic venous ulcers, hypertension, CVI, obesity, childhood-onset type conduct disorder, and cognitive communication deficit. His family history and social history are noncontributory. On physical examination, the patient was alert and in no apparent distress. Bilateral lower extremities revealed severe hyperkeratosis along with nodular skin growths resembling “tree bark” skin changes (Figure 1). An ulcer measuring 7 × 6 cm on the left posterior ankle was examined, which had a purulent discharge and was located above the calcaneus (Figure 2). In addition, multiple small ulcers at different stages of healing were noted on bilateral ankles and feet. Capillary refill was less than two seconds with no surrounding crepitations, and peripheral pulses were palpated bilaterally. At the time, the patient denied any pain, and there was no tenderness to palpation on bilateral lower extremities.

In the ED, the patient’s old dressing was removed, and the wounds were left open to air. His vital signs were as follows: blood pressure, 159/67 (normal: 120/80); temperature (oral), 97.8°F (normal: 97-99°F); and heart rate, 78 bpm (normal: 60-100 bpm). The laboratory workup performed at the emergency department is listed in Table 1.

**Table 1:** Laboratory investigations performed at the time of initial presentation in the emergency department

Test	Result	Reference range
Hemoglobin	10.6 g/dL	11-13.7 g/dL
Hematocrit	34.2%	34%-44%
White blood cells	6.6 × 10^9^/L	4.5-11 × 10^9^/L
Creatinine	1.35 mg/dL	0.3-1.35 mg/dL
Point-of-care glucose	98 mg/dL	70-100 mg/dL
Venous lactate	1.5 mmol/L	0.5-2.2 mmol/L

**Figure 3:**
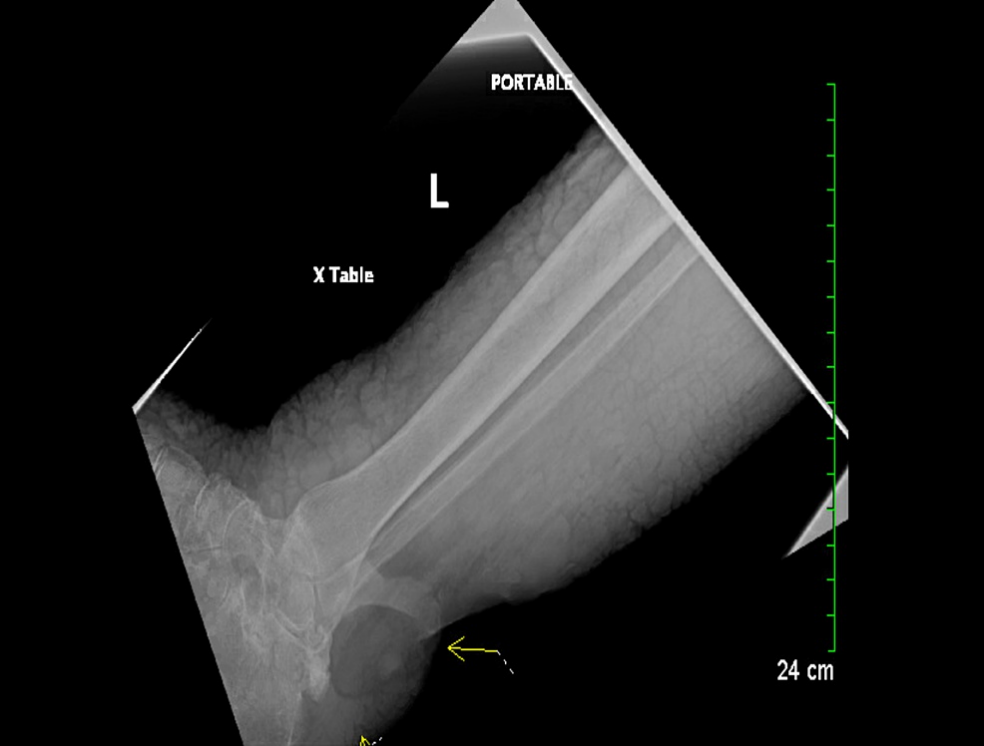
X-ray imaging demonstrating a large ulceration in the soft tissues overlying the posterior aspects of the left ankle

X-ray imaging demonstrated a large ulceration in the soft tissues overlying the posterior aspects of the left ankle (Figure 3) without any evidence of bony resorptive changes or periosteal reaction bilaterally, thus ruling out necrotizing fasciitis and osteomyelitis. Surgery was consulted thereafter, and the patient was admitted to the hospital for a surgical wound debridement of a necrotic ulcer on the left ankle. In accordance with infectious disease (ID) recommendations, blood cultures and wound drainage cultures were then obtained, and the patient was started on prophylactic intravenous (IV) vancomycin and cefazolin with close monitoring of creatinine levels. Syphilis and human immunodeficiency virus (HIV) screening tests yielded negative results. As per wound/ostomy care recommendations, alginate dressing was applied to the patient’s ulcers, urea cream was applied to bilateral lower extremities, and compression wraps were used to reduce any swelling. Cardiology was then consulted to further evaluate the patient’s hypertension and CVI, and IV heparin was initiated prophylactically to prevent deep vein thrombosis (DVT). Bilateral venous duplex ultrasound yielded negative results for DVT, and computed tomography (CT) angiogram revealed bilateral lower extremity superficial reflux with enlargement of bilateral greater saphenous veins and numerous superficial varicosities below the calf soft tissues along the greater saphenous vein courses bilaterally. The patient remained hemodynamically stable, and blood pressure was controlled with amlodipine.

On the fourth day of admission, surgical debridement of the left ankle ulcer was performed without any intraoperative complications. Operative cultures were obtained, and the patient was still closely monitored. On first post operative day (POD 1), he reported mild left lower extremity pain, for which acetaminophen was administered. The patient was stable and afebrile. Wound care treatment with collagenase Santyl (Smith and Nephew, London, UK) application was resumed, along with heel elevation and moisture barrier cream application. On second post operative day, wound drainage culture and operative cultures were positive for *Proteus mirabilis* superinfection. Vancomycin and cefazolin were discontinued at this time, and oral cefdinir treatment was initiated. The patient was discharged back to the NH on the fifth post operative day and advised to follow up with outpatient wound care. The patient has not been readmitted in-patient as of the time of writing.

## Discussion

Venous insufficiency (VI) is a prevalent condition in the adult population, especially in western countries. Studies have demonstrated that the prevalence of venous insufficiency can range anywhere from <1% to 40% in females and <1%-17% in males [8]. There are many risk factors associated with venous insufficiency, including age, female gender, pregnancy, family history of venous disease, obesity, smoking, and occupations associated with orthostasis [8]. The majority of cases are typically asymptomatic depending on the severity of the VI. However, in some patients, it may lead to serious complications. Most of these manifestations are dermatologic and are mainly due to venous hypertension and lymphedema secondary to chronic venous insufficiency. These are dermatologic changes that can occur with CVI, such as atrophie blanche, cellulitis, cutaneous fungal infections, fibromas, fissures, folliculitis, hyperkeratosis, ingrown toenails, intertrigo, lymphorrhea, lymphocyst, malignancies, pachydermia, papillomatosis cutis lymphostatica, pressure ulcerations, stasis dermatitis, toe and foot deformities, and venous stasis ulceration with superinfection. Patients with VI are more likely to develop contact dermatitis as well. This is most likely due to disturbance in the epidermal barrier, increasing the likelihood of sensitization against certain allergens [4]. The symptoms and clinical presentation of stasis dermatitis include brown discoloration, leg swelling and heaviness, skin ulcers, thickening and bumpiness of the skin, and crusting or cracking of the skin. These patients may also present with pain and itchiness. In the context of superinfection, an increase in white blood cell count, fever, chills, and increasing pain can be noted.

**Figure 4:**
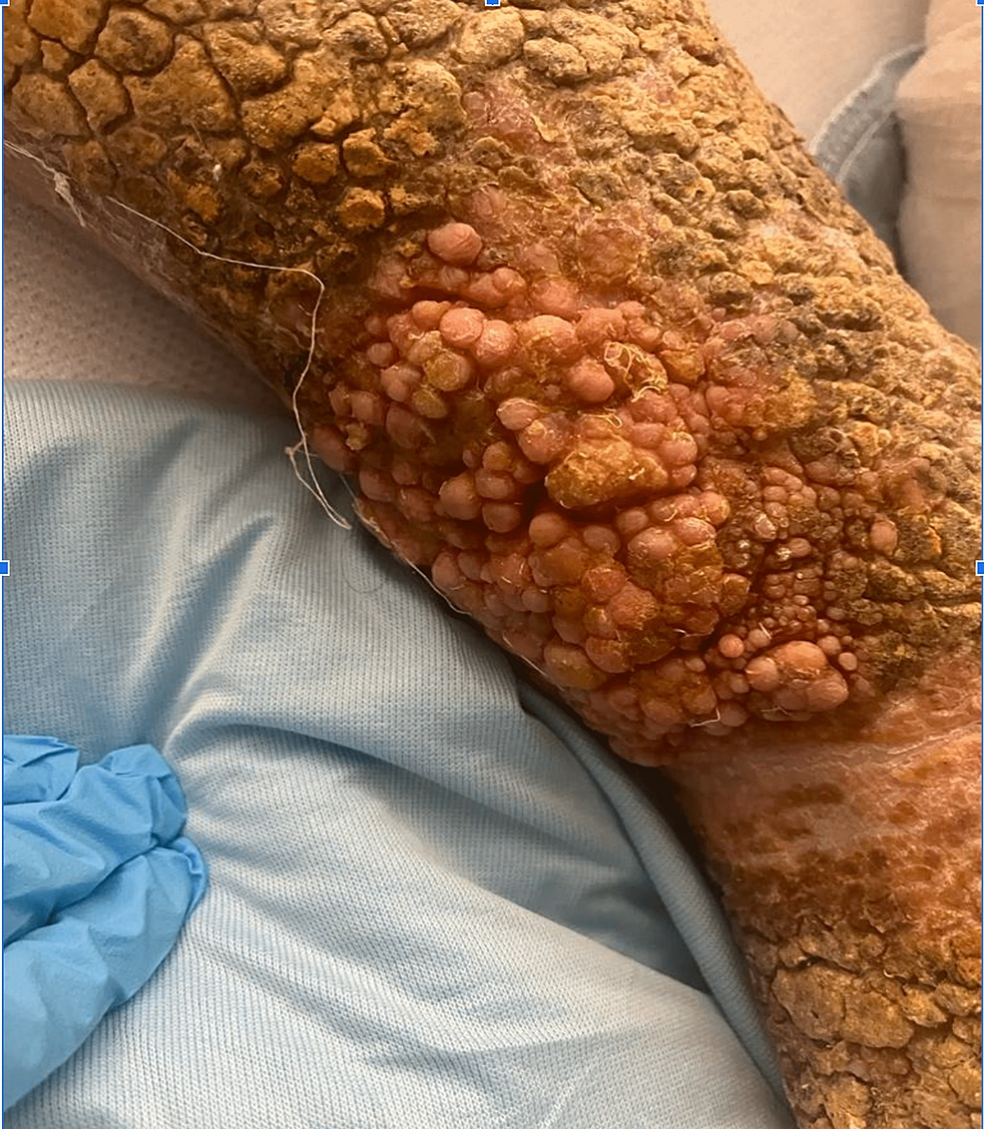
Papillomatosis cutis lymphostatica manifesting as verrucous papillomatous lesions along with lymphedema

If VI is left untreated, as was the case in our patient, there is a possibility for the emergence of severe complications. These include hyperkeratosis and skin ulcers prone to infections. In our patient, we had a severe dermatologic presentation of venous insufficiency that included skin ulcer with superinfection (Figure 2), papillomatosis cutis lymphostatica that is benign but an otherwise very unusual complication of CVI, lymphedema (Figure 4) [9], and hyperkeratosis (Figure 1). Other observable findings include ingrown toenails and pachydermia (Figure 1 and Figure 4). Aydin and Heidenheim explained in their case findings that papillomatosis cutis lymphostatica can be a result of combinations of lymphedema, CVI, and diabetes, all of which our patient possessed.

The diagnostic workup usually begins with a Doppler ultrasound of arterial and venous flow. In the case of VI, this would usually demonstrate decreased venous flow, and if the patient is diabetic, as in our case, arterial flow can be compromised as well. Since our patient came in with a painful skin ulcer with concomitant infection, we had an X-ray performed to rule out any involvement of the bone and surrounding deep tissues for osteomyelitis and necrotizing fasciitis, respectively (Figure 3). The draining infection seen in Figure 2 was swabbed and sent to the laboratory for culture. The causative organism was found to be *Proteus mirabilis*, which is not uncommon in these cases.

Moreover, one may elect to perform genetic testing to rule out rarer conditions such as epidermodysplasia verruciformis (aka “tree man syndrome”) that mimic our patient’s dermatologic findings. This is a genetic condition that affects the normal immunologic function of the skin and predisposes individuals to severe human papillomavirus (HPV) manifestations [10]. Other hyperkeratotic skin conditions should also be considered in the differential diagnosis. These include but are not limited to callus and corns, keratosis plantare, chronic folliculitis, atopic dermatitis, psoriasis and psoriasiform dermatitis, lichen planus and dermatitis, keratosis pilaris, ichthyoses, seborrheic keratosis, actinic keratosis, keratoacanthoma, paraneoplastic syndromes, squamous cell carcinoma, and basal cell carcinomas [11]. To differentiate these, skin biopsy and thorough family history may be warranted.

To be able to treat and adequately manage dermatologic problems associated with VI, the underlying issues of edema and VI must be addressed. In addition, modifiable risk factors should be minimized. Therefore, steps to treatment include minimizing standing for long periods of time, elevating legs while sitting, and reducing body weight [12]. Compression stockings or medicated leg wraps can aid in preventing blood pooling in the extremities and reducing venous pressure [12]. Smoking cessation should be encouraged, as smoking can have detrimental effects on venous valves. Symptoms such as itching and dryness can be alleviated using hypoallergenic moisturizers or petroleum-based ointments. On occasion, medicated steroid creams can be prescribed to help reduce skin inflammation or itch [12]. The prophylactic use of topical antibiotics such as neomycin or bacitracin is not recommended as it can lead to the development of skin allergy. These patients are already at a higher risk of developing allergic reactions due to the destruction of normal skin barriers [12]. The treatment of papillomatosis cutis lymphostatica is similar, consisting of topical ointments, compression, and surgical excision. If skin ulcers with superinfections occur, surgical management in the form of debridement is recommended, as was completed in our patient. To prevent recurrent ulcers, it is highly recommended to increase physical activity, exercise, mobility, and leg elevation [13]. Moreover, there is also significant evidence on compression therapy substantially decreasing the risk of recurrence [13]. Although these recommendations can aid in reducing significant symptoms, it is not a definitive cure and the majority of patients typically return with similar symptoms and ulcer manifestations.

## Conclusions

In conclusion, we presented a case of a 67-year-old male with CVI and lymphedema with papillomatosis cutis lymphostatica, hyperkeratosis, and skin ulcers with proteus superinfection. This case presentation highlights the importance of prompt intervention, vigilant monitoring, and, overall, adequate long-term management of CVI. Poorly managed CVI may lead to complications such as lymphedema, venous hypertension, venous ulcerations, hyperkeratosis, and secondary skin infection, as was seen in our case. Furthermore, the cutaneous finding of papillomatosis cutis lymphostatica is a severe complication that may mimic other dermatologic conditions and thus requires a good level of diagnostic suspicion on the basis of the patient’s present and prior medical history.
